# *De Novo* Transcriptome Analysis of *Allium cepa L*. (Onion) Bulb to Identify Allergens and Epitopes

**DOI:** 10.1371/journal.pone.0135387

**Published:** 2015-08-18

**Authors:** Hemalatha Rajkumar, Ramesh Kumar Ramagoni, Vijayendra Chary Anchoju, Raju Naik Vankudavath, Arshi Uz Zaman Syed

**Affiliations:** 1 Department of Microbiology & Immunology, National Institute of Nutrition, Indian Council of Medical Research, Hyderabad, Telangana State, 500 007, India; 2 Biomedical Informatics Center, National Institute of Nutrition, Indian Council of Medical Research, Hyderabad, Telangana State, 500007, India; 3 Nucleome Informatics Pvt. Ltd., Hyderabad, Telangana State, 500 049, India; Cankiri Karatekin University, TURKEY

## Abstract

*Allium cepa* (onion) is a diploid plant with one of the largest nuclear genomes among all diploids. Onion is an example of an under-researched crop which has a complex heterozygous genome. There are no allergenic proteins and genomic data available for onions. This study was conducted to establish a transcriptome catalogue of onion bulb that will enable us to study onion related genes involved in medicinal use and allergies. Transcriptome dataset generated from onion bulb using the Illumina HiSeq 2000 technology showed a total of 99,074,309 high quality raw reads (~20 Gb). Based on sequence homology onion genes were categorized into 49 different functional groups. Most of the genes however, were classified under 'unknown' in all three gene ontology categories. Of the categorized genes, 61.2% showed metabolic functions followed by cellular components such as binding, cellular processes; catalytic activity and cell part. With BLASTx top hit analysis, a total of 2,511 homologous allergenic sequences were found, which had 37–100% similarity with 46 different types of allergens existing in the database. From the 46 contigs or allergens, 521 B-cell linear epitopes were identified using BepiPred linear epitope prediction tool. This is the first comprehensive insight into the transcriptome of onion bulb tissue using the NGS technology, which can be used to map IgE epitopes and prediction of structures and functions of various proteins.

## Introduction

*Allium cepa* (onion) is one of the most important vegetable crops in the *Allium* family known for its nutritional and medicinal properties [1], [2]. Onion perhaps is among the earliest cultivated vegetables and dates back to 5000 years [3]. Onions have antioxidant, anti-cholesterol anticancer properties and anti-inflammatory activitydue to phenolics compounds and flavonoids; but can induce symptoms of food allergy such as asthma, rhino conjunctivitis and contact dermatitis in sensitised individuals [4–7]. Diallyl disulphide is a major allergen in garlic and onion that is known to cause contact dermatitis [1], [8].

In food allergic individuals, exposure to allergenic epitopes from food proteins causes production of specific IgE antibodies that bind to the surface of tissue bound mast cells or circulating basophils, which ultimately releases inflammatory mediators. The actions of these mediators cause the clinical signs and symptoms of food allergy [9], [10]. Quite a few diagnostic techniques are available for the diagnosis of IgE mediated food allergy such as double-blind placebo-controlled food challenge (DBPCFC), skin prick test (SPT), specific IgE antibody test, case history etc. [11], [12]. However, diagnosis of food allergy is complicated with low or no detectable levels of specific IgE in patients with symptoms of IgE mediated food allergy [13], [14] and detection of specific IgE is not necessarily associate with clinical symptoms [15–17]. Novel diagnostic methods such as focusing on protein and epitope specificity are under investigation. In this scenario, epitopes on onion bulb allergens were predicted as onion is widely consumed and is known to cause IgE mediated allergy [1], [18–20].

*A*. *cepa* is a diploid (2n = 16) plant and has approximately 16.4 giga (billion) bases per 1C; it's nuclear genome is one of the largest nuclear genomes among all diploids and is over six times greater than maize or humans [21], [22]. Though onion has been used extensively in the past for cytogenetic studies, molecular analysis has been lacking.

Although modern sequencing and proteomic technologies permit the ready detection of numerous protein sequence variants in any organisms, for onions, there are no allergenic proteins and genomic data available. Onion is an example of an under-researched crop which has a complex heterozygous genome. Whereas genome based research has previously been hindered by limited sequence resources and allergenic protein information.

In recent years several studies have successfully reported the generation of transcriptome data and its analysis as an effective tool to study gene expression in specific tissues and also provide a platform to address comparative genomics for gene discovery in non-model plants in which no reference genome sequences are available [23–25]. Next-generation sequencing (NGS) technologies, such as Illumina, Genome Sequencer FLX system (GS FLX), and ABI SOLiD, allow analysis of the transcriptome because of increased throughput and reduced sequencing cost [25–29]. Due to the availability of quick, low cost sequencing and high quality annotation using different assembly tools, it has become possible to analyze and understand the transcriptome of onion plant [30].

This study describes the generation, *de novo* assembly and annotation of transcriptome dataset derived from cDNAs obtained from onion bulb using the Illumina HiSeq 2000 sequencing technology. The final assembly was functionally annotated, allowing for the identification of putative genes responsible for allergenicity and other similar sequences. Identification of these genes leads to testable hypotheses concerning their conserved function and to rational strategies to improve allergen free (genetically modified) onions.

## Materials and Methods

### Plant material

Fresh onion bulb (Pusa Madhavi light red onion variety) was collected from our institutional vegetable garden and the bulb was cut into small pieces in RNA later solution to extract RNA.

### RNA isolation and quality control

Total RNA was isolated using the High Pure RNA Isolation Kit (Roche Life sciences,Basel, Switzerland) following the manufacturer’s instructions. The integrity was checked with bio-analyzer (Agilent 2100 Technologies, USA) to yield high quality RNA for further processing. The mRNA was extracted and purified from the total RNA by using TruSeq stranded mRNA HT sample preparation kit (Illumina Inc., U.S.A), followed by purity check with Qubit fluorometer before proceeding to cDNA synthesis.

### Paired-end library preparation and Illumina sequencing

The cDNA was synthesized and libraries were prepared with the above-mentioned TruSeq Stranded mRNA HT Sample prep kit according to manufacturer's protocol. After PCR amplification the libraries were purified on agarose gel. The quality of the library was assessed by Qubit 2.0 fluorometer (Life technologies, USA.) and inserts with 150–250 bp were selected. Clustering was performed using TruSeq PE cluster kit v3-cBot-HS on cBot (Illumina Inc, USA). The samples were run (2x100) on Illumina HiSeq 2000 instrument using TruSeq SBS kit v3-HS (200 cycles) (Illumina Inc, USA) following manufacturer's instructions. The sequence data generated in this study have been deposited at NCBI, Sequence Read Archive (SRA) database under the accession number PRJNA248253 (BioProject) with experiment accession number SRX547958 for PE reads.

### Raw data pre-processing and *de novo* transcriptome assembly

The sequenced raw data was processed to obtain high quality clean reads, we used the open-source software package Trimmomatic v0.32 to identify and to trim nucleotides falling below the established quality threshold (minimum 20 phred score) as well as to trim adapter sequences [31]. A minimum length of 50 nt (nucleotide) after trimming was applied. Orphaned reads were assigned as single-end reads. Onion bulb whole transcriptome assembly was carried out in three different stages. At first stage of assembly, the high quality clean reads were assembled by using Velvet (Version 1.2.10) followed by Oases (Version 0.2.8) to built k-mer specific transcripts from k-mer 27 to k-mer 63 (S1 Table), both Velvet and Oases are meant for assembly of short reads [32], [33]. The second stage of assembly was performed to obtain merged assembly of transcripts obtained from first stage i.e., from k-mer 27 to k-mer 63 using Velvet and Oases long read option. The third/final stage of assembly was performed by using CD-HIT which produces a set of 'non-redundant' (nr) onion bulb transcriptome catalogue [34]. The GC content analysis of onion transcripts was performed by using in-house perl scripts.

### Similarity search and functional annotation

To deduce the putative function, onion transcripts were used as queries to search protein databases using BLASTx (Basic Local Alignment Search Tool) programme against the NCBI non-redundant (nr) green plant database, homology searches were performed with an e-value cut-off of 1E-06. Gene Ontology (GO) classification was carried out by using BLASTx results in Blast2GO software for functional classification of GO terms [35]. GO terms were obtained from nr hits using Blast2GO software with default parameters during mapping and an e-value cut-off of 1E-06 was used for BLAST hits in the BLAST2GO annotation step.

### Identification of allergens and homologous sequences

For the identification of food and other allergens and their families represented in onion transcriptome, the onion transcripts were searched against the allergen data collected using BLASTx with an E-value cut-off of 1E-05 and the default parameters for BLASTx were used: Expected value is 10, word size is 3, scoring matrix is BLOSUM62, gap penalty existence is 11, gap penalty extension is 1 and "low complexity region" was not selected.

Total number of 553 allergen groups which has protein sequences were collected from the allergen data source, AllergenOnline, version 11, 1491 sequences (553 groups, 265 species) which is maintained by Food allergy research and resource programme (FARRP) allergen protein sequences, University of Nebraska–Lincoln [36]. For allergen identification contiguous 80 amino acid sequence length transcripts were separated. E values and identity percentages were evaluated to estimate potential cross-reactivity that may endow with an immunological target for IgE antibodies.

### Identification of epitopes on allergenic proteins

Contigs that had shown best E-Values in the BLASTx top hits were considered for epitope prediction. Accelrys Discovery Studio Visualizer (version 4.0) was used to translate contigs in nucleotide sequences to protein sequences [37]. These translated protein sequences were submitted to antibody epitope prediction tool by "BepiPred linear epitope prediction" method [38]. Internally BepiPred use Parker hydrophilicity scale, Levitt secondary structure scale, and Hidden Markov Models [39–41]. The default values were maintained for threshold (0.350) and window size (7). The predicted epitope length with start and end positions were mentioned including residue scores.

## Results

### Sequencing and *de novo* transcriptome assembly to build onion transcriptome catalogue

A total of 99,074,309 high quality raw sequence reads (~20Gb) were generated using Illumina HiSeq 2000 technology (Table 1). The raw data filtration was carried out by using Trimmomatic v0.32 which resulted in high quality 83,046,820 paired-end (PE), 1,384,023 single-end forward and 93 single-end reverse reads (Table 2). After quality assessment and data filtering, the high quality clean reads were used for *de novo* assembly of the onion bulb transcriptome in three different stages. In the first stage of assembly, the high quality clean reads were assembled using Velvet/Oases assembler at different k-mer lengths (S1 Fig, S1 Table). At second stage of assembly, we merged the transcripts which were obtained from first stage of assembly into a consensus transcriptome using Velvet and Oases long read option (S2 Table). The non-redundant (nr) representative sequences were generated by the CD-HIT algorithm at the third and final stage of assembly (S2 Table), which resulted in 293,475 transcripts of 280,882,036 bp in size. On analyzing the final assembly only transcripts with more than 100 bp length were considered for functional annotation (S2 Fig). GC content (Guanine and Cytosine ratio) of the onion bulb transcriptome catalogue was determined using in-house perl script and the average GC content was found to be 38% (Table 2).

**Table 1:** Summary of raw reads of *Allium cepa* bulb transcripts.

Raw reads	99,074,309 (~20Gb)
Clean PE reads	83,046,820
Clean SE forward reads	1,384,023
Clean SE reverse reads	93
G+C%	43%

Gb, Giga bases; PE, paired end; SE, single end; G+C, Guanine+Cytocine; %, percentage

**Table 2:** Statistics of non-redundant set of *Allium cepa* bulb transcripts obtained from final stage of assembly.

Total number of transcripts	293,475
G+C%	38%
Total transcriptome length	280,882,036 bp
Average transcript size	957.1 bp
Transcript N50	1,594 bp
Max. transcript size	12,638 bp

G+C, Guanine+Cytocine; %, percentage; Max, maximum; bp, base pairs

### Functional annotation and characterization of onion transcripts

**Fig 1:**
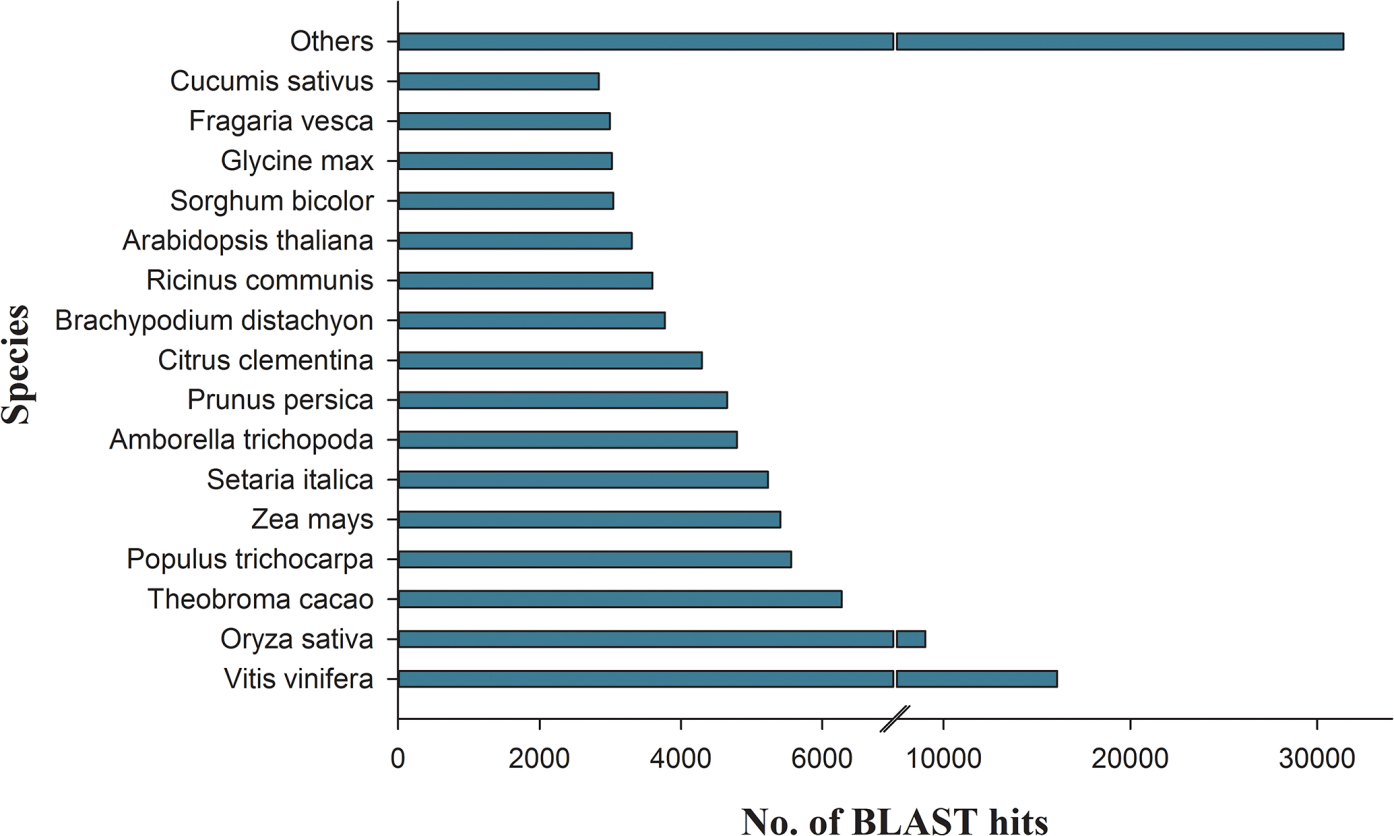
Annotations and BLAST top hits of onion transcripts with other species. This figure shows species distribution of onion transcripts with other species by BLASTx. Most of the onion sequences were homologous to *Vitis vinifer*a (14%) followed by *Oryza sativa* (8%) and *Theobroma caca*o (5.4%).

The assembled onion bulb transcript set was analysed for similarity/sequence conservation against the NCBI green plant nr-database using BLASTx search. To define a significant hit, an E-value cut-off threshold of ≤1E-06 was considered. From the total of 293,475 onion transcripts, 15,434 transcripts were annotated by BLASTx search and 4,780 transcripts were mapped against nr database by using BLAST2GO tool. In addition, of the 293,475 transcripts, a total of 115,251 transcripts had unique matches, whereas 178,224 transcripts had no blast hits (S3 Fig). Overall, a total of 115,251 (40%) transcripts exhibited significant similarity with at least one of the predicted protein from sequenced plants in the database. Likewise, we analysed the sequence conservation of onion transcripts with proteomes of all sequenced plant species. From the 115,251 transcripts, the highest matching of onion transcripts was observed with *Vitis vinifera* (14%) followed by *Oryza sativa* (8%), *Theobroma cacao* (5.4%) and other species (Fig 1). However, only 1,086 (1%) sequences were found as onion specific.

### Functional classification of onion transcriptome by GO

In order to assign putative functions, transcripts from onion were compared against the GenBank’s nr-protein sequences of green plants and UniProt database using BLASTx algorithm. The associated hits were searched for their respective GO. Based on sequence homology *Allium cepa* genes were categorized into 49 different functional groups and were categorized as biological process, cellular component and molecular function (S4 Fig). Most of the genes however, were classified under ‘unknown groups’ in all three GO categories. Of the categorized genes, 61.2% showed metabolic functions followed by other functions and cellular components such as binding (58.5%), cellular processes (57.6%), catalytic activity (47.7%) and cell and cell part (45.6%).

### Identification of allergens & epitopes of onion transcripts with allergen database

**Fig 2:**
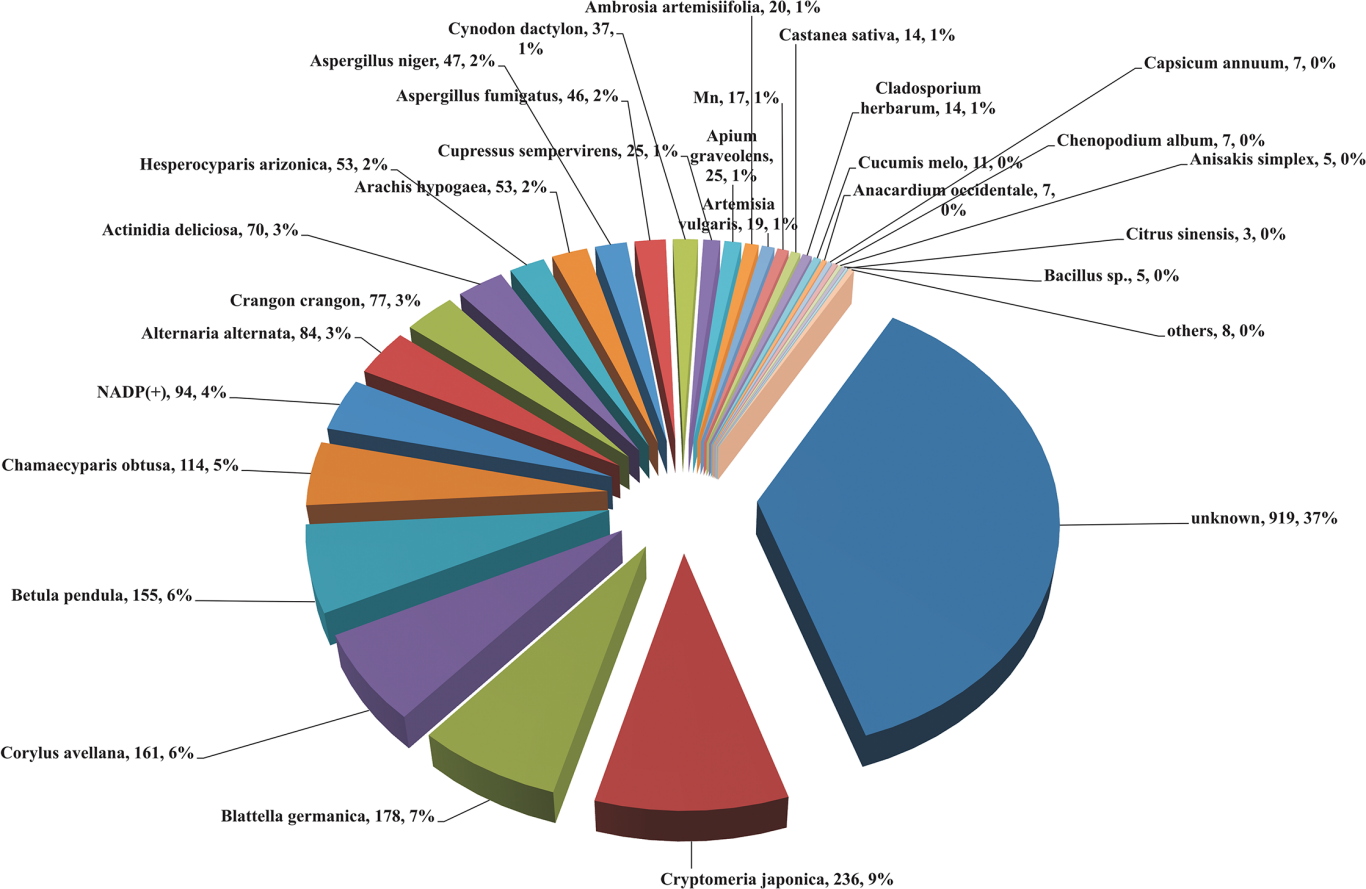
Onion allergen species distribution identified with BLASTx. The data represents number of transcripts and percentage of species distribution of onion bulb. For each species that was matching with onion, total number of hits is given along with relative percentage of homology. The comma (,) is separating the number of transcritpts from the % of species distribution. Highest number of onion transcripts have shown homology to *Cryptomeria japonica* allergens (9%), followed by *Blattella Germania* (7%) and *Corylus avellana* (6%).

**Fig 3:**
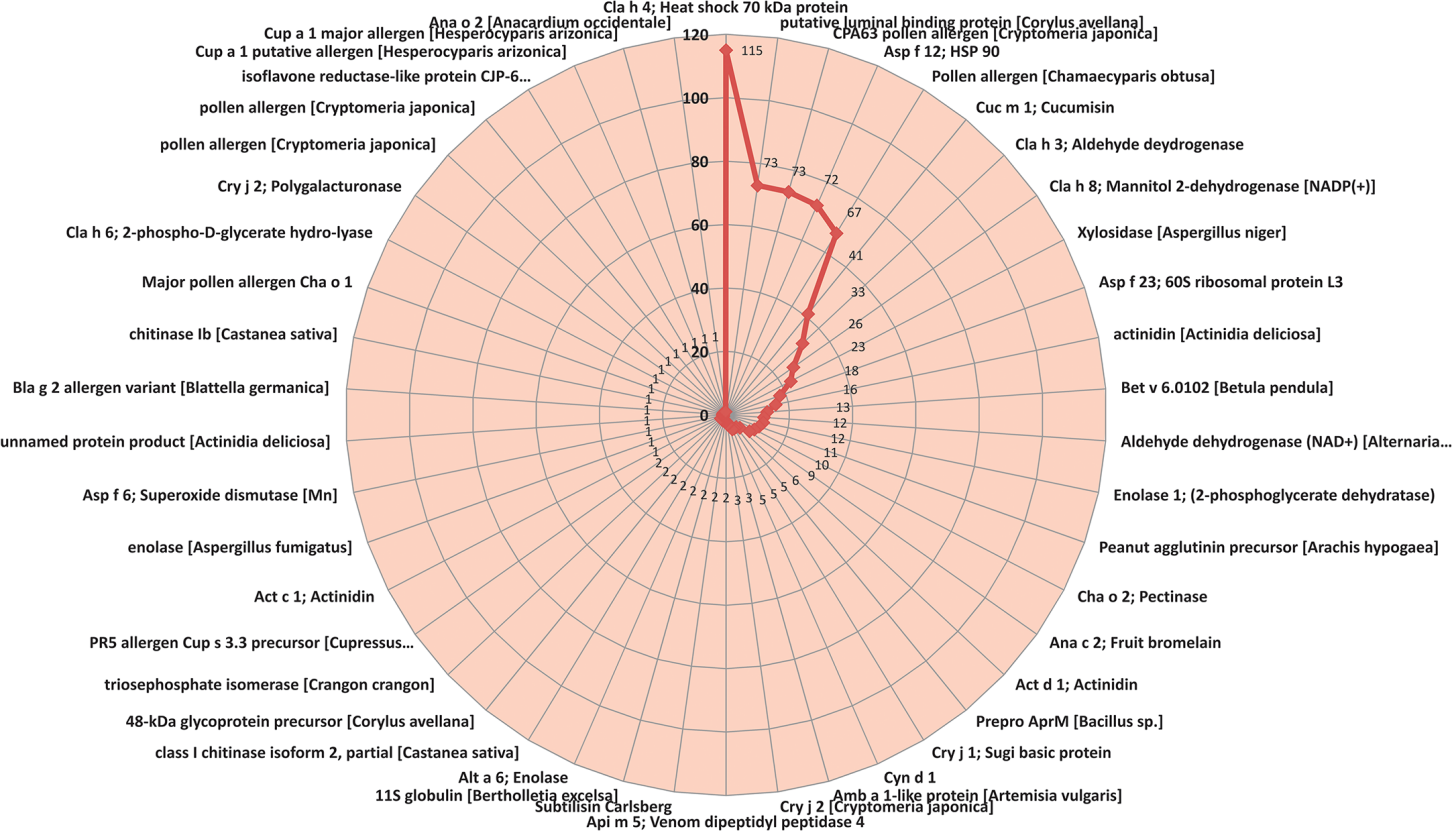
Onion allergens identified through transcriptome analysis. This radar graph depicts onion allergens identified through transcriptome analysis. Highest number of transcripts were homologous to Cla h4 (115) followed by putative luminal binding protein of *Corylus avellana* (73), CPA 63 pollen allergen *Cryptomeria japonica* (73), and Asp f12 of fungi (72). (Selected transcript sequence size: ≥80 amino acids).

Onion allergen species distribution was identified with BLASTx top hits. Most of the onion transcripts were categorized under “unknown species” against allergen database. A total of 2,511 homologous allergenic sequences were found in onion transcriptome, which had 37 to 100% similarity with existing allergen database. A huge number of onion transcripts have shown homology to *Cryptomeria japonica* (Japanese cedar, 9%) allergens, followed by *Blattella Germania* (German cockroach, 7%) and *Corylus avellana* (Common hazel, 6%) (Fig 2). Of the 2,511 homologous allergenic sequences identified, when nucleotide bases of 240 or more (equal to 80 amino acid length or more) were considered for blast top hits, 682 transcripts from onion bulb were found to be similar to published allergen sequences, which were altogether 46 different types. Thus, 46 allergens were identified from onion bulb whole transcriptome (Fig 3).

From the BLASTx results the onion transcripts contig 116485 showed E-value of ‘0’, bit-score of 525.013 and homology of 75% with Enolase 1, 2-phospho-D-glycerate hydro-lyase. The onion contig 165540 showed E-value of 4.93E-169, bit-score of 483.411 and homology of 76% with ASP f 23 allergen (Table 3). From the 46 identified allergens (BLASTx results) epitopes were predicted using BepiPred linear epitope prediction tool. A total of 521 B cell linear epitopes were identified from 46 allergens of onion bulb when amino acid length of six or more was considered (S3 Table).

**Table 3:** The E-values and homology (%) of *Allium cepa* transcripts with matched allergen sequences.

Sequence Name	Allergen	Biochemical Function	Species	Common name	Type	Homology (%)	Seq. Len	Align. Len	E-Value	Bit-Score
Contig116485	N/A	Enolase 1, 2-phospho-D-glycerate hydro-lyase	N/A	N/A	N/A	75	1889	438	0	525.013
Contig165540	Asp f 23	60S ribosomal protein L3	Aspergillus fumigatus	A. Fumigatus	Fungi	76	1596	417	4.93E-169	483.411
Contig124037	Asp f 12	65 kDa IgE-binding protein; Heat shock protein hsp1	Aspergillus fumigatus	Fungi	Fungi	67	3117	680	1.46E-159	487.263
Contig147925	Alt a 11, Alt a 6, Alt a 5	Enolase; 2-phosphoglycerate dehydratase	Alternaria alternata	Fungi	Fungi	69	1980	431	9.71E-158	460.685
Contig147926	N/A	AF284645_1 enolase	Aspergillus fumigatus	Fungi	Fungi	69	1850	413	3.42E-149	437.187
Contig134658	Cuc m 1	Cucumisin, serine protease	Cucumis melo	Muskmelon	Plant	56	2397	735	9.67E-135	416.001
Contig121132	N/A	Xylosidase	Aspergillus niger	A. Niger	Fungi	53	2776	762	2.29E-133	418.313
Contig122480	N/A	Actinidin	Actinidia deliciosa	Fuzzy Kiwifruit	Plant	70	2423	360	2.09E-125	379.793
Contig192886	Cup a 1	Putative allergen Cup a 1	Hesperocyparis arizonica or Cupressus arizonica	Arizona cypress	Plant	63	1515	355	1.13E-111	334.339
Contig149472	N/A	Chitinase Ib	Castanea sativa	Sweet chestnut	Plant	74	1181	306	2.04E-111	327.791
Contig181997	N/A	Amb a 1-like protein	Artemisia vulgaris	Mugwort	Plant	63	1798	370	3.06E-110	334.724
Contig147924	Cla h 6	Enolase; 2-phospho-D-glycerate hydro-lyase; 2-phosphoglycerate dehydratase	Cladosporium herbarum	Fungi	Fungi	69	1739	303	1.22E-108	331.643
Contig185872	Cry j 1	Sugi basic protein	Cryptomeria japonica	Japanese cedar	Plant	61	1653	361	9.33E-108	326.25
Contig213065	N/A	Unnamed protein product	Actinidia deliciosa	Fuzzy Kiwifruit	Plant	67	1172	319	1.39E-107	320.087
Contig239367	N/A	11S globulin	Bertholletia excelsa	Brazil nut	Plant	61	1679	449	1.21E-104	321.627
Contig105897	Cha o 1	Major pollen allergen Cha o 1	Chamaecyparis obtusa	Japanese cypress	Plant	65	1915	355	3.22E-102	314.309
Contig87206	Act d 1	Actinidain	Actinidia deliciosa	Kiwi	Plant	66	1513	329	6.81E-102	309.686
Contig165488	Ana c 2	Fruit bromelain	Ananas comosus	Pineapple	Plant	65	1227	333	7.57E-100	300.056
Contig82715	Bet v 6.0102	Allergenic isoflavone reductase-like protein Bet v 6.0102	Betula pendula	Silver birch	Plant	68	1364	306	4.57E-99	298.13
Contig134058	CJP-6	Isoflavone reductase-like protein CJP-6	Cryptomeria japonica	Japanese cedar	Plant	64	1279	312	7.41E-97	291.197
Contig88117	N/A	Triosephosphate isomerase	Crangon crangon	Common shrimp	Crustacean	70	2141	248	3.05E-89	278.1
Contig97550	Cha o 2	Polygalacturonase; Major pollen allergen Cha o 2; Pectinase	Chamaecyparis obtusa	Japanese cypress	Plant	53	1956	466	1.48E-85	275.789
Contig181817	Cry j 2	Allergen Cry j 2	Cryptomeria japonica	Japanese cedar	Plant	60	1769	358	2.34E-84	270.781
Contig70943	Cla h 4	Heat shock 70 kDa protein	Cladosporium herbarum	Fungi	Fungi	48	3004	645	5.05E-84	281.952
Contig116658	Cla h 10	Aldehyde dehydrogenase; Cla h 3	Cladosporium herbarum	Fungi	Fungi	50	2328	483	5.37E-60	207.608
Contig141069	N/A	Pollen allergen	Cryptomeria japonica	Japanese cedar	Plant	47	1672	313	2.23E-59	155.221
Contig128098	N/A	Class I chitinase isoform 2	Castanea sativa	Sweet chestnut	Plant	56	1528	247	7.32E-58	191.815
Contig116297	Act c 1	Actinidain	Actinidia deliciosa	Kiwi	Plant	50	1678	323	6.34E-55	187.193
Contig16398	Cup s 3.3 precursor	PR5 allergen Cup s 3.3 precursor	Cupressus sempervirens	Pencil pine or Mediterranean cypress	Plant	57	1445	241	2.31E-54	179.489
Contig122189	Asp f 6	Superoxide dismutase [Mn]	Aspergillus fumigatus	Fungi	Fungi	50	1281	241	3.89E-53	174.481
Contig86874	N/A	Aldehyde dehydrogenase (NAD+)	Alternaria alternata	Fungi	Fungi	45	2257	462	9.06E-53	186.422
Contig70938	N/A	Putative luminal binding protein	Corylus avellana	Common hazel	Plant	44	4214	721	4.39E-52	191.045
Contig152875	CPA63	Pollen allergen CPA63	Cryptomeria japonica	Japanese cedar	Plant	48	1590	437	6.92E-52	180.644
Contig175155	Api m 5	Venom dipeptidyl peptidase 4	Apis mellifera (Honeybee)	Honeybee	Insect	43	2585	622	8.42E-42	158.303
Contig176760	Cup a 1	Major allergen Cup a 1	Hesperocyparis arizonica or Cupressus arizonica	Arizona cypress	Plant	49	1577	253	2.10E-39	142.124
Contig198455	N/A	48-kDa glycoprotein precursor	Corylus avellana	Common hazel	Plant	44	1792	400	3.56E-36	135.191
Contig122991	Ana o 2	Legumin-like protein	Anacardium occidentale	Cashew tree	Plant	42	1378	401	2.51E-31	119.398
Contig178413	Cyn d 1	Major pollen allergen Cyn d 1	Cynodon dactylon	Bermuda grass	Plant	47	1150	242	7.30E-26	99.3673
Contig154078	N/A	Pollen allergen	Chamaecyparis obtusa	Japanese cypress	Plant	40	1895	435	9.82E-26	104.375
Contig181406	N/A	Subtilisin Carlsberg	Bacillus sp.	Bacillus	Bacteria	44	3643	246	1.49E-22	95.5153
Contig163717	N/A	Pollen allergen	Cryptomeria japonica	Japanese cedar	Plant	44	1663	260	4.98E-20	87.4261
Contig140011	N/A	Peanut agglutinin precursor	Arachis hypogaea	Peanut	Plant	48	2216	273	8.64E-19	81.6481
Contig155816	Bla g 2 allergen	Bla g 2 allergen variant	Blattella germanica	German cockroach	Insect	46	1842	240	3.19E-16	74.3294
Contig189186	Cla h 8	Mannitol 2-dehydrogenase [NADP(+)]	Cladosporium herbarum	Fungi	Fungi	43	1355	278	3.56E-15	69.3218
Contig157948	Cry j 2	Major pollen allergen; Polygalacturonase; Pectinase	Cryptomeria japonica	Japanese cedar	Plant	41	1014	249	1.47E-14	68.1662
Contig144878	Apr M	Prepro Apr M	Bacillus sp.	Bacillus	Bacteria	43	4317	253	1.08E-09	55.8398

Contig, Contiguous sequence; Seq. Len, Sequence length; Align. Len, Alignment length; %, percentage; E-value, Expect value; NA, not applicable

## Discussion

Onion is one of the most important vegetable crops in the *Allium* family, known for its nutritional and medicinal properties. Vegetables of the genus *Allium* are widely consumed, but remain poorly understood genetically. *Allium* species are notable for their very large genomes, and typically ranges from10–20 Gbp, which have made it difficult for genomic studies and therefore have precluded genome sequencing to date [42].

Very few genomic resources including EST sequences and molecular markers are available for onion bulb compared to other vegetable species [43]. Transcriptome catalogue of onion bulb was generated for the first time, using which allergens and epitopes were identified in the current study. Transcriptome analysis is particularly useful for revealing relationships between plant gene expression and phenotypes. To build the transcriptome, the sequencing lengths of 2×100 bp paired-end sequencing was opted using Illumina HiSeq 2000 V3 chemistry, which is suitable for analysis of 200–5,000-bp lengths. This system is the most efficient and accurate approach to demarcate the boundaries of transcription units of genes and complements other methods for transcriptome studies [44].

In this study, Velvet and Oases assemblers were used in the first two stages for assembly of short read sequences. This technique yielded long N50 lengths and therefore we could obtain better quality assembly of short reads. An earlier study by Rohini G et al. also showed the *de novo* assembly by Velvet followed by Oases programmes and validated these programs for better assembly [45]. In the final stage of assembly we had used CD-HIT algorithm to reduce the redundancy.

Nearly 100 million sequence reads were generated for onion bulb in the current study and 293,475 non-redundant set of transcripts were found (Tables 1 and 2). Only few transcripts were annotated, and complete information on gene functionality could be obtained for few genes. Interestingly, half of the amount of onion transcripts did not show significant homology with existing sequences, suggesting novel genes that may perform species specific functions in onion bulb; nevertheless, from the functional annotation a large number of onion transcripts showed significant similarity with predicted proteins of plants. Examining a non-model vegetable plant such as onion can provide novel insights into the underlying mechanisms and diversity, however, to our surprise, only 1,086 (1%) sequences were identified as onion (*Allium cepa*) specific, thus signifying that limited information is available about the genomes or transcriptomes of onion and its related species. In the present study a large number of transcripts from *Allium cepa* were classified as unknown, suggesting the existence of massive potential for new gene identification.

Gene Ontology is an international standardized gene functional classification system that offers a strictly defined concept to comprehensively describe the properties of genes and gene products in any organism [35]. By using GO tool, we obtained a high number of onion bulb genes that were classified under either cell and cell part (cellular component) or metabolic processes (biological process) or binding activity indicating the dominance of genes that are responsible for cellular processes and metabolism, gene regulation and transcription factor binding processes (S4 Fig).

Base composition is a fundamental property of genomes and has a strong influence on various aspects related to gene function, regulation, structure (intron size and number), thermo-stability and species ecology [46–48]. However, the biological significance of GC content diversity in plants remains unclear because of a lack of sufficiently robust genomic data. The average GC content of the onion bulb transcripts in the present study was ~38%, which is very similar to what has been reported previously (Table 2) [49].

Whole trascriptome sequencing may change the ways in which gene expression is studied, which is likely to have much future impact. The growing number of available allergen sequences together with the advancements of bioinformatics tools and methods will enable us to shed light on evolutionary and structural relationships between allergens from different sources [50].

Using bioinformatics approach we compared the onion bulb transcripts with existing allergen sequences in databases such as AllergenOnlline, FARRP, Allergome and Structural database for allergenic proteins (SDAP) to predict potential cross reactivity of onion bulb transcriptome sequences with available database [51], [52]. In our transcriptome data we have found more than 2,500 homologous sequences to 553 existing allergen groups containing 1,491 sequences of 265 different species. According to guidelines of Food and Agriculture Organisation/World health organization (FAO/WHO) and Codex (2003) the sequences which show more than 35% identity against amino acid sequences of expressed proteins (allergens) are considered as cross reactive allergens (>80 amino acid transcript sequences) [53]. By adopting these guidelines we have identified 682 homologous sequences and 46 different allergens in onion bulb. Transcript sequences generated from this study can be used to map epitopes of monoclonal antibodies and polyclonal sera from patients. With the support of total transcriptome of onion, the complete list of genes can be predicted based on which unknown protein structures may be modelled and novel diagnostic approach in food allergy and immunotherapeutic can be developed. Compared to the previously described random peptide libraries, the transcriptome RNA gene sequencing as performed in the current study offers good approach to identify epitopes. More than 500 B cell linear epitopes were identified from onion bulb when amino acid length of six or more was considered.

## Conclusion

From the 99,074,309 high quality raw reads that were generated from onion bulb, 49 different functional groups of genes were identified. With BLASTx top hit analysis, a total of 2,511 homologous allergenic sequences were found, which had 37 to 100% similarity with 46 different types of allergens existing in the database. From the 46allergens, more than 500 B-cell linear epitopes were identified. This is the first comprehensive insight into the transcriptome of onion bulb tissue using the NGS technology, which can be used to map IgE epitopes and prediction of structures and functions of various proteins.

## Supporting Information

S1 Fig *De novo* assembly of onion bulb transcriptome. This figure represents the *De novo* assembly of onion bulb transcriptome generated by Velvet/Oases (1^st^ and 2^nd^ stage) and by CD-HIT (3^rd^ stage). The bars indicate number of contigs (100 bp or longer). The lines indicate N50 length in bp (light green colour line with triangles) and average contig length (dark red colour line with rectangles). The left Y- axis indicates number of contigs and the right Y- axis indicates length in bp.
(TIF)

S2 Fig *De novo* assembly length distribution. Frequency histogram showing number of transcripts as function of onion assembly read length distribution. The highest sequence length was 12,635 bp and only transcripts with ≥100 bp length sequences were considered for functional annotation.
(TIF)

S3 Fig Onion BLASTx statistics. This figure shows the BLAST hits results including annotations and mapping.
(TIF)

S4 Fig Histogram of onion Gene Ontology classification. GO terms obtained and classified into three major groups as Cellular Component, Molecular Function and Biological Process. Most of the onion GO terms observed as cell and cell part, metabolic processes, catalytic and binding processes.
(TIF)

S1 Table Stage one onion (*Allium cepa L*.) bulb transcriptome assembly statistics. In stage 1 assembly Velvet/Oases tools were used to assemble high quality reads to generate kmer specific transcripts.
(XLS)

S2 Table Assembly statistics of onion (*Allium cepa* L.) bulb transcriptome using Velvet/Oasis and CD-Hit tools. This table represents the statistics of assembled transcripts at second and third stages of assemblies.
(DOC)

S3 Table The allergenic peptide or epitope prediction. The 46 identified allergens (BLASTx results) from onion bulb showed a total of 521 B cell linear epitopes when amino acid length of 6 or more was considered.
(XLS)
