# Supplementary figures and images for: De Novo Transcriptome Analysis of Allium cepa L. (Onion) Bulb to Identify Allergens and Epitopes
Source: PLoS One. 2015 Aug 18;10(8):e0135387. doi: 10.1371/journal.pone.0135387 (PMC4564285; doi:10.1371/journal.pone.0135387)

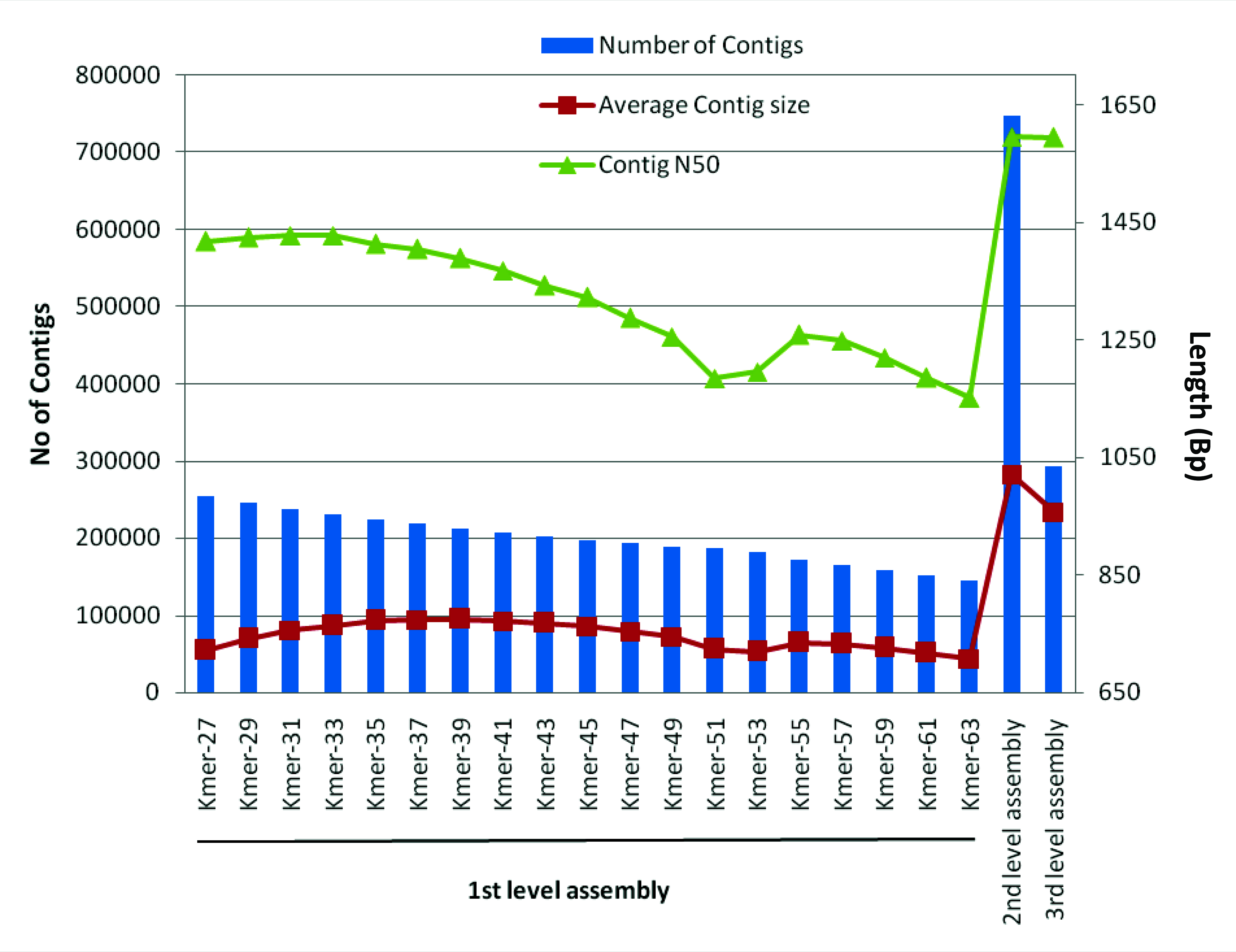

Supplement: S1 Fig — This figure represents the De novo assembly of onion bulb transcriptome generated by Velvet/Oases (1st and 2nd stage) and by CD-HIT (3rd stage). The bars indicate number of contigs (100 bp or longer). The lines indicate N50 length in bp (light green colour line with triangles) and average contig length (dark red colour line with rectangles). The left Y- axis indicates number of contigs and the right Y- axis indicates length in bp. (TIF) [file pone.0135387.s001.tif]

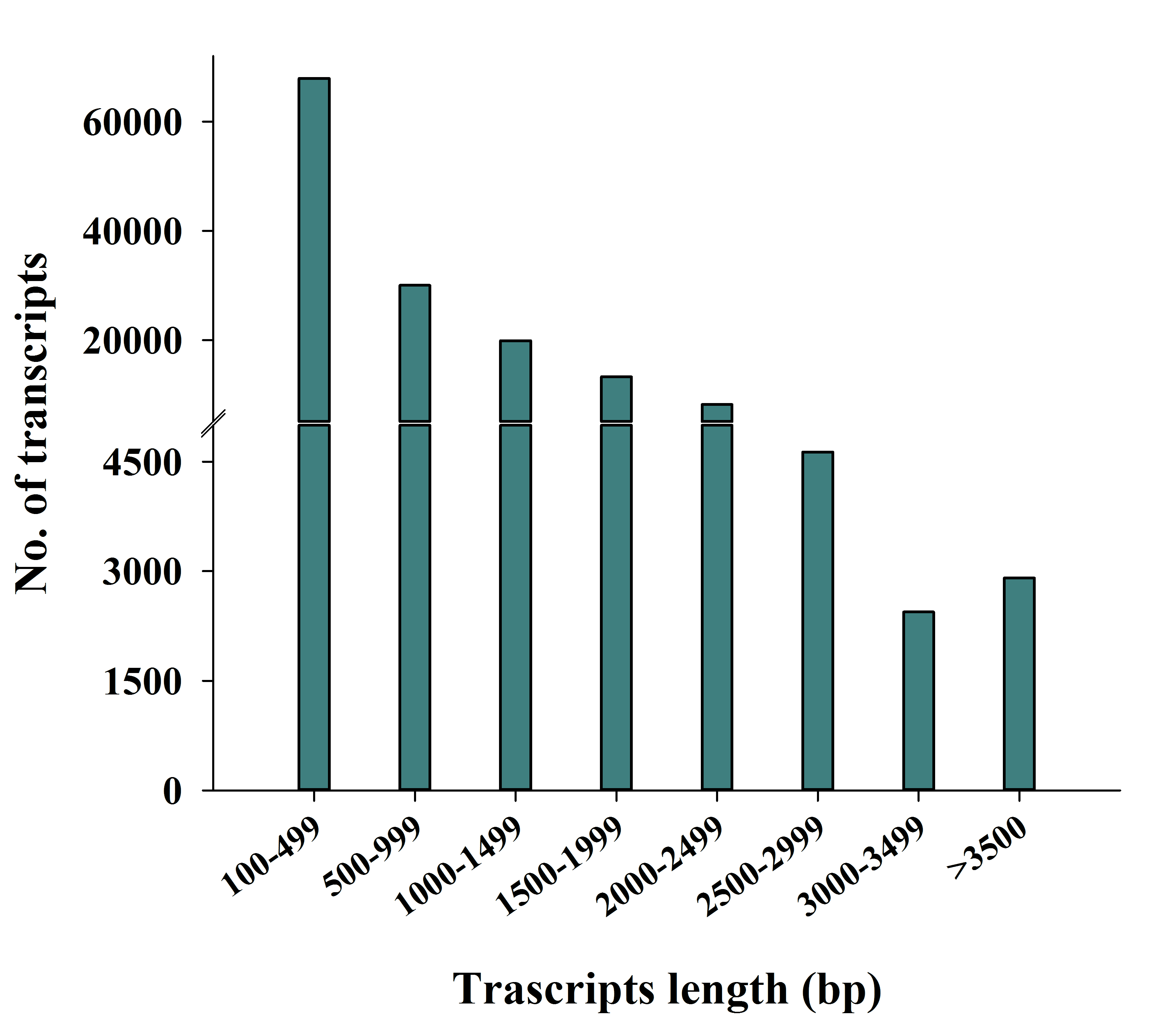

Supplement: S2 Fig — Frequency histogram showing number of transcripts as function of onion assembly read length distribution. The highest sequence length was 12,635 bp and only transcripts with ≥100 bp length sequences were considered for functional annotation. (TIF) [file pone.0135387.s002.TIF]

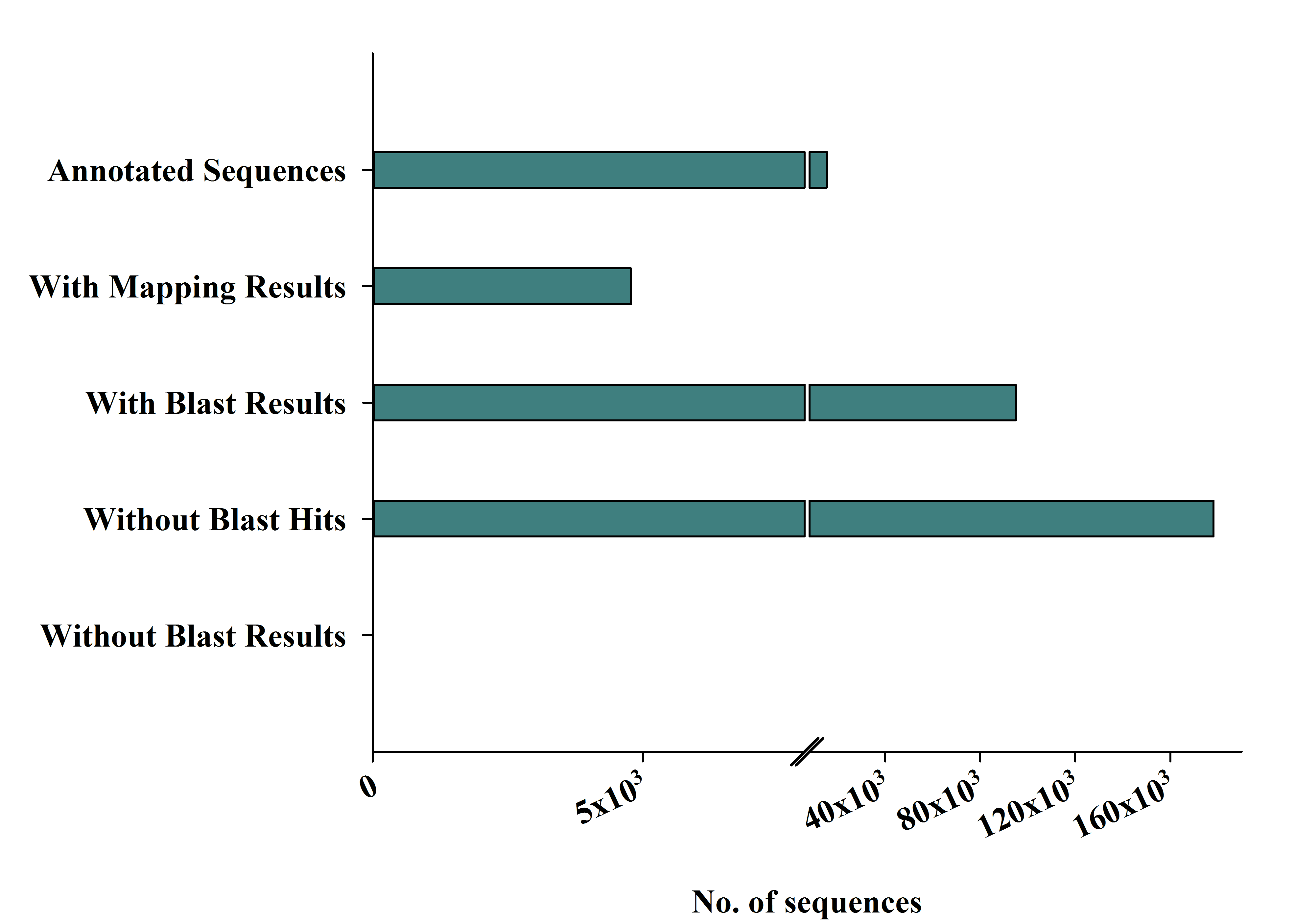

Supplement: S3 Fig — This figure shows the BLAST hits results including annotations and mapping. (TIF) [file pone.0135387.s003.TIF]

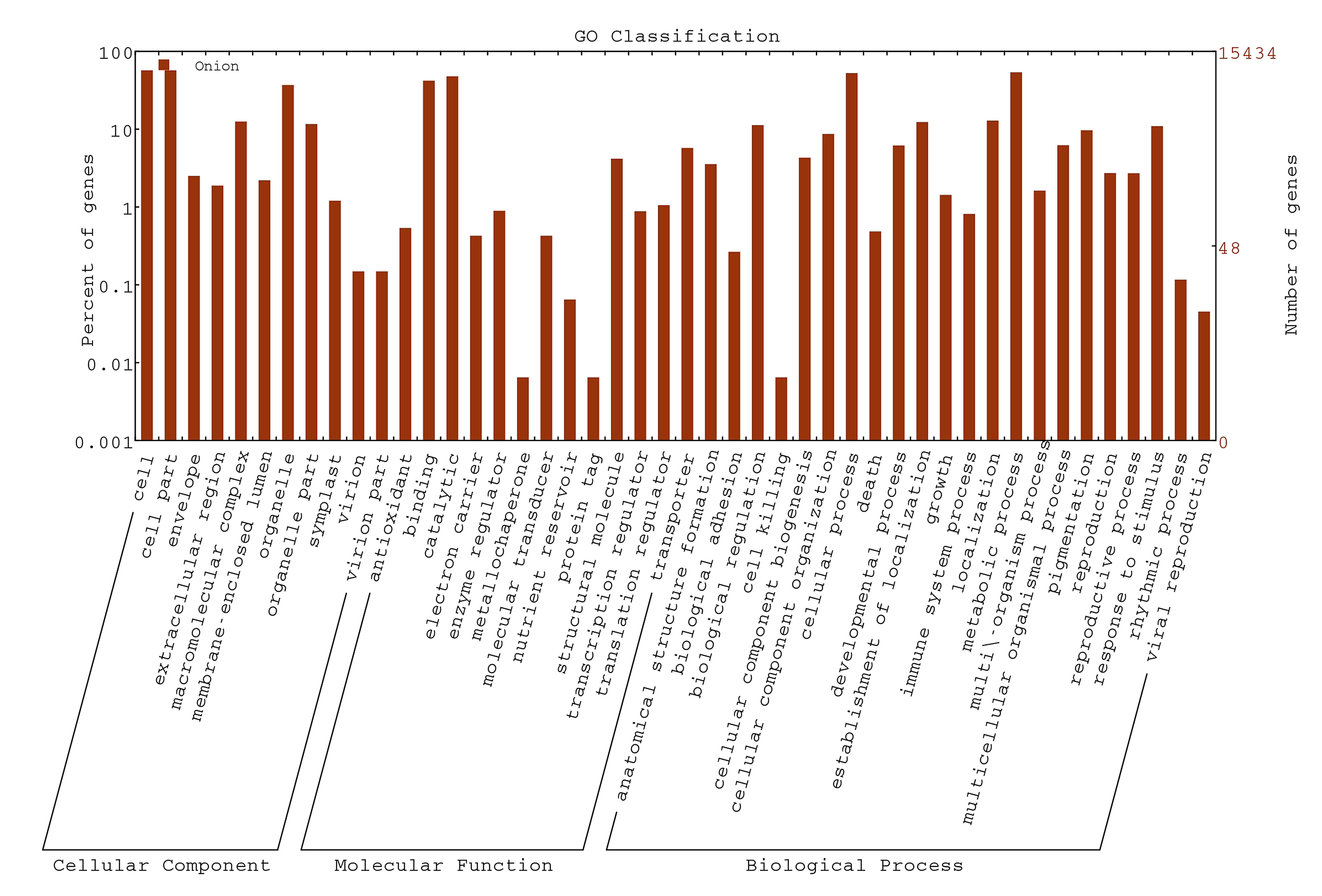

Supplement: S4 Fig — GO terms obtained and classified into three major groups as Cellular Component, Molecular Function and Biological Process. Most of the onion GO terms observed as cell and cell part, metabolic processes, catalytic and binding processes. (TIF) [file pone.0135387.s004.tif]
