# Supplementary material for: De Novo Transcriptome Analysis of Allium cepa L. (Onion) Bulb to Identify Allergens and Epitopes
Source: PLoS One. 2015 Aug 18;10(8):e0135387. doi: 10.1371/journal.pone.0135387 (PMC4564285; doi:10.1371/journal.pone.0135387)
Supplement: S2 Table — This table represents the statistics of assembled transcripts at second and third stages of assemblies. (DOC) [file pone.0135387.s006.doc]

**S2 Table.** **Assembly statistics of Onion (*Allium cepa* L.) bulb transcriptome using Velvet/Oasis and CD-Hit tools.** This table represents the statistics of assembled transcripts at second and third stages of assemblies.

|  | **Second stage of assembly** | **Third stage of assembly** |
| --- | --- | --- |
| Number of scaffolds | 746788 | 293475 |
| Number of contigs | 746788 | 293475 |
| Total genome length including gaps | 762138882 | 280882036 |
| Total genome length without gaps | 762138882 | 280882036 |
| Average scaffold size including gaps | 1020.555877 | 957.0901644 |
| Average scaffold size without gaps | 1020.555877 | 957.0901644 |
| Averaging contig size | 1020.555877 | 957.0901644 |
| Contig N50 | 1595 | 1594 |
| Scaffold N50 | 1595 | 1594 |
| Maximum scaffold size | 12638 | 12638 |
| Minimum scaffold size | 100 | 100 |

The N50, maximum, minimum and average contig and scaffold sizes were given for only the contigs ≥100 bp.
